# Characterisation of airway inflammation and proteomes associated with cystic fibrosis-related diabetes

**DOI:** 10.1183/23120541.00290-2025

**Published:** 2025-11-10

**Authors:** Stefanie Diemer, Sounak Chowdhury, Cecilia Sahl, Lotta Happonen, Lisa I. Påhlman

**Affiliations:** 1 Skåne University Hospital, Department of Paediatrics, Lund, Sweden; 2 Wallenberg Centre for Molecular Medicine, Lund, Sweden; 3 Lund University, Department of Clinical Science Lund, Division of Infection Medicine, Lund, Sweden; 4 Skåne University Hospital, Division of Infectious Diseases, Lund, Sweden

## Abstract

**Background:**

Cystic fibrosis (CF)-related diabetes (CFRD) is the most common extrapulmonary complication in CF. CFRD is associated with low lung function, but the underlying mechanisms are poorly understood. The aim of the present study was to compare airway inflammation and the airway proteome in people with CF (pwCF) with and without CFRD.

**Methods:**

Sputum samples from pwCF were analysed for neutrophil elastase (NE) activity with a chromogenic assay, inflammatory cytokines using Meso Scale and bacterial load *via* quantitative PCR of the 16S rRNA gene. The sputum proteome was characterised by liquid chromatography-mass spectrometry.

**Results:**

33 pwCF were included in the study, of which 55% had CFRD. The CFRD group had significantly lower lung function and higher sputum levels of NE, interleukin (IL)-8 and IL-1β, whereas IL-6 levels were lower compared to pwCF without CFRD. Proteome analysis identified 27 sputum proteins linked to CFRD, mainly involved in neutrophil degranulation. Given that lung function could be a possible confounding factor, we matched pwCF with and without CFRD based on lung function. In these lung function-matched cohorts, IL-8 and IL-6 levels did not differ significantly, but IL-1β showed a trend towards higher levels in the CFRD group. 10 CFRD-associated proteins were significantly more abundant in the CFRD group, including prothymosin α, which plays a role in diabetes and insulin release.

**Conclusion:**

CFRD is associated with lower lung function, increased sputum levels of NE, IL-8 and IL-1β, and specific protein profiles.

## Shareable abstract

**CF-related diabetes is a common complication in CF and is associated with a decline in lung function. This study found increased airway inflammation and specific sputum protein expression patterns in patients with CF-related diabetes.** https://bit.ly/3EnkoZg

## Introduction

Cystic fibrosis (CF) is the most common life-threatening inherited disorder in the Caucasian population. CF is caused by mutations in the CF transmembrane conductance regulator (*CFTR*) gene [1], which encodes a chloride and bicarbonate channel. Pulmonary disease characterised by chronic infections, chronic inflammation and subsequent lung function decline is a hallmark of CF. In the airways, expression of defective *CFTR* causes viscous secretions and impaired mucociliary clearance leading to microbial colonisation and inflammation. However, the CFTR channel is also expressed in other organs, including the gastrointestinal tract, the reproductive organs and pancreas.

CF-related diabetes (CFRD) is the most common extrapulmonary complication of CF and is associated with increased morbidity and mortality [2]. The prevalence of CFRD increases with age, affecting 1–2% of children, around 20% of adolescents and approximately every third adult with CF [3, 4]. It is well recognised that CFRD is tightly linked to low lung function and impaired nutritional status [5, 6]. People with CFRD (pwCFRD) have a higher rate of pulmonary exacerbations and worse lung disease compared to people with CF (pwCF) [7]. The exact mechanisms underlying lung function decline in CFRD is poorly understood, but are likely multifactorial [8, 9]. The impaired glucose homeostasis with hyperglycaemic peaks in the airways could affect airway epithelial cells, promote bacterial growth and increase oxidative stress [10–12]. Previous studies have demonstrated increased serum inflammatory markers in pwCFRD [13] and increased inflammation and airway destruction in murine models of CFRD [14, 15], suggesting that inflammatory pathways are involved in the association between CFRD and low lung function. To further investigate airway inflammation and CFRD-related lung function decline, more *in vivo* studies are needed.

In the present study, we hypothesised that impaired glucose control would increase airway inflammation. We therefore aimed to characterise inflammation and protein expression patterns in sputum samples from pwCF with and without CFRD.

## Material and methods

### Study population

All study participants were prospectively recruited at the CF centre of Skåne University Hospital Lund, Sweden, during 2021 and 2022. Inclusion criteria were age ≥18 years, a typical clinical presentation of CF and diagnosed disease-causing mutations in the *CFTR* gene. Exclusion criteria were a history of organ transplantation, inability to expectorate a sputum sample, ongoing airway exacerbation or missing oral glucose tolerance test (OGTT) screening within the last 12 months for patients without an already established CFRD diagnosis.

OGTT was performed according to a standardised protocol as described before [16]. CFRD was defined as a fasting blood glucose ≥7 mmol·L^−1^ and/or a 120-min glucose value ≥11.1 mmol·L^−1^ [17]. HbA1c was measured at baseline.

Clinical data were collected from the Swedish CF registry and from medical journals. Forced expiratory volume in 1 s in per cent of predicted (FEV_1_ % pred) was measured using the Global Lung Function Initiative equation [18]. Chronic infection with *Pseudomonas aeruginosa* was defined as growth of *P. aeruginosa* in ≥50% of airway cultures according to Leeds criteria [19].

The study was approved by the local Medical Ethics Committee in Lund (reference number 2018/54 with amendments 2021-05475-02 and 2023-06075-02). Written informed consent was obtained from all participants.

### Sputum sample collection and preparation

Study participants donated an induced sputum sample to the study at the time of their annual clinical review. The sputum sample collection was supervised by a physiotherapist.

Sputum samples were liquefied with 0.1% dithiothreitol (DTT) (Sigma-Aldrich) as described before [20]. One aliquot of the liquefied sputum was stored at −80°C for later DNA extraction. The remaining sample was centrifuged at 1000×g for 10 min, and the cell-free supernatant was collected and stored at –80°C until further analysis.

### Nucleic acid extraction and quantitative PCR

Total DNA was extracted from 200 µL of liquefied sputum using bead-beating with Pathogen Lysis Tubes S (Qiagen ref.19091) followed by QIAamp UCP Pathogen Mini Kit (Qiagen ref.50214) according to the protocol of the manufacturer. Total bacterial load was assessed with quantitative PCR of the bacterial 16 s rRNA gene using primers described previously [21]. Sputum samples were diluted 1:1000 in nuclease-free H_2_O, and 5 µL template was added to 15 µL mastermix containing iTaq Universal SYBRGreen Supermix (Bio-Rad), primers and nuclease-free H_2_O. Known concentrations of *P. aeruginosa* DNA in 10-fold dilutions were analysed in parallel and used as a standard.

### Neutrophil elastase activity

Neutrophil elastase activity in cell-free sputum samples was measured using the chromogenic substrate N-methoxysuccinyl-Ala-Ala-Pro-Val p-nitroanilide (Sigma Aldrich) as described before [20]. After incubation at 37°C for 30 min, the absorbance at 405 nm was measured. All samples were analysed in duplicate.

### Quantification of inflammatory cytokines

Cytokine levels in cell-free sputum samples were analysed using Meso Scale immunoassays (Meso Scale Diagnostics LLC, Rockville, MD, USA) according to the protocol provided by the manufacturer. Tumour necrosis factor-α (TNF-α), IL-6 and IL-1β were analysed in a multiplex U-PLEX with a sample dilution of 1:5. IL-8 was analysed using U-PLEX and a sample dilution of 1:100, and IL-17 was quantified with S-PLEX at a 1:5 dilution. All samples were analysed in duplicate.

### Statistical analysis

Statistical analyses were performed using GraphPad Prism 10.0.2 software (GraphPad Software, San Diego, CA, USA). Comparisons between groups were made using Mann–Whitney U-test. Fisher's exact test was used to compare categorical values. A p-value ≤0.05 was determined as statistically significant.

### Preparation and data independent acquisition liquid chromatography-tandem mass spectrometry of sputum samples

For mass spectrometry (MS) analysis, 10 μL of DTT-treated, cell-free sputum samples were prepared as described [22]. For data-independent acquisition (DIA) liquid chromatography-mass spectrometry (LC-MS/MS) analysis, 1 μL of peptides was analysed on an Orbitrap Eclipse mass spectrometer connected to an ultra-high-performance liquid chromatography Dionex Ultra300 system (both Thermo Scientific). The peptides were loaded and concentrated on an Acclaim PepMap 100 C18 precolumn (75 μm × 2 cm) and separated on an Acclaim PepMap RSLC column (75 μm × 25 cm, nanoViper, C18, 2 μm, 100 Å; both columns Thermo Scientific), at a column temperature of 45°C and a maximum pressure of 900 bar. A linear gradient of 2–25% of 80% acetonitrile (can) in aqueous 0.1% formic acid (FA) was run for 100 min followed by a linear gradient of 25–40% of 80% ACN in aqueous 0.1% FA for 20 min. For DIA, one full MS scan (resolution 120 000; mass range of 350–1650 m/z) was followed by MS/MS scans (resolution 30 000). The precursor ions fragmented using higher-energy collisional-induced dissociation (HCD) at a normalised collision energy of 30. For DIA, 44 variable isolation windows were used.

### DIA data analysis

DIA data were analysed by the direct DIA workflow by using Spectronaut (version 18.4.231017.55695) against the UniProt reviewed human database containing 20 418 entries. Default settings were applied for peptide and protein identification and quantification. Fully tryptic digestion was used allowing two missed cleavages. Carbamidomethylation (C) was set to static and oxidation (M) as well as protein N-terminal acetylation to variable modifications. The number of identifications was controlled by false discovery rate (FDR) of 1% at peptide and protein level, respectively.

The resulting datasets were analysed in Perseus (version 2.0.9.0), including proteins identified by two or more peptides. The label-free quantification (LFQ) intensity values were log2 transformed, and mean transformed missing values were imputed from a Gaussian distribution (width=0.2 and downshift=1.8). For volcano plots, differentially enriched proteins in the groups compared were identified by two-sample t-test (FDR<0.05, s0=1). Gene enrichment analyses were performed using Metascape [23]. All MS data have been deposited to the ProteomeXchange consortium *via* the MassIVE partner repository https://massive.ucsd.edu/ with the dataset identifier PXD064131.

## Results

### Patient characteristics

A total of 40 pwCF were enrolled in the study. Seven participants were later excluded due to organ transplantation (n=1) and uncertain glucose tolerance status (n=6), resulting in a final cohort of 33 included pwCF. Sputum collection from pwCF without a known CFRD diagnosis was done in connection with OGTT, except for seven participants who performed OGTT within 12 months prior to sampling (range 8–12 months).

Of the total cohort, 18 participants (55%) had a CFRD diagnosis and 13 (72%) of pwCF with CFRD were treated with insulin. Demographic and clinical data for the included participants are shown in table 1. 20 patients (61%) were on treatment with a *CFTR* modulator, mainly lumacaftor-ivacaftor. Only six patients received triple modulator therapy, as this treatment was only available through a compassionate use programme or *via* clinical trials at the time of inclusion. HbA1c was significantly increased in pwCF with CFRD, suggesting an impaired glycaemic control. In addition, the CFRD group had a significantly lower lung function (table 1).

**TABLE 1:** Patient characteristics

Demographic and clinical data	All patients	PwCF without CFRD	PwCF with CFRD	p-value
**Patients, n**	33	15	18	
**Age, years, median (IQR)**	38 (27–39)	32 (25–39)	39 (29–40)	0.27
**Female, n (%)**	10 (30)	5 (33)	5 (28)	>0.99
**Homozygous ΔF508, n (%)**	18 (55)	9 (60)	9 (50)	0.73
**BMI, kg·m^−2^, median (IQR)**	23 (21–25)	24 (21–24)	23 (20–27)	0.88
**FEV_1_ % pred, median (IQR)**	71 (54–86)	86 (70–96)	60 (45–74)	<0.01
**Chronic *P. aeruginosa*, n (%)**	22 (67)	8 (53)	14 (78)	0.16
**Pancreatic insufficiency, n (%)**	32 (97)	14 (93)	18 (100)	0.45
**Treatment with *CFTR* modulator, n (%)**	20 (61)	10 (67)	10 (56)	0.72
**HbA1c, mmol·mol^−1^, median (IQR)**	41 (36–45)	37 (34–41)	43 (41–50)	<0.001

PwCF: people with cystic fibrosis; CFRD: cystic fibrosis-related diabetes; BMI: body mass index; FEV_1_: forced expiratory volume in 1 s.

### CFRD is associated with increased sputum inflammation

**FIGURE 1:**
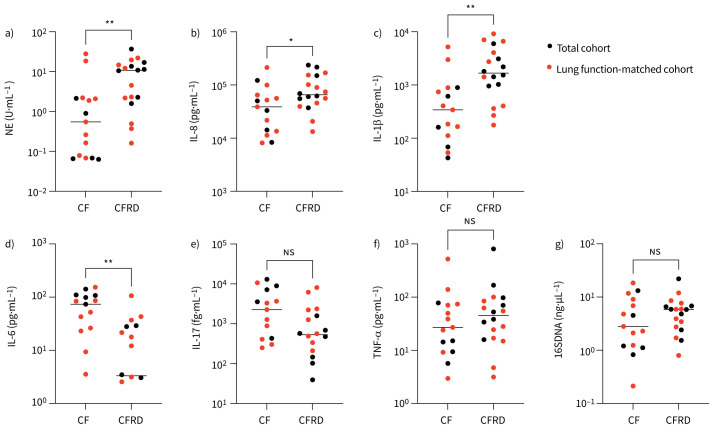
Neutrophil elastase (NE) activity and expression of inflammatory cytokines in cystic fibrosis (CF) sputum. Sputum samples from people with CF with (n=18) or without CF-related diabetes (CFRD) (n=15) were analysed for NE activity using a) a chromogenic assay, and the inflammatory cytokines b) interleukin (IL)-8, c) IL-1β, d) IL-6, e) IL-17 and f) tumour necrosis factor-α (TNF-α) using Meso Scale U-PLEX assays, and g) bacterial DNA using qPCR of the 16SDNA gene. Red dots represent patients matched on lung function. Bars represent median values. NS: not significant. *p<0.05; **p<0.01.

To compare airway inflammation between pwCF with and without CFRD, neutrophil elastase (NE) activity and inflammatory cytokines were quantified in sputum samples from the study participants. NE activity was significantly higher among patients with CFRD, indicating a higher neutrophil burden in the lower airways (figure 1a). Similarly, airway concentrations of interleukin (IL)-8 and IL-1β were significantly higher in the CFRD group (figure 1b and c). Conversely, IL-6 levels were lower in pwCF with CFRD (figure 1d). There were no statistically significant differences between the groups when comparing the expression of IL-17 and TNF-α (figure 1e and f). Moreover, sputum concentrations of bacterial DNA were similar between the groups (figure 1g).

Previous work has demonstrated a negative association between airway inflammation and lung function [24], and the differences in FEV_1_ % pred between pwCF with and without CFRD in this study could therefore confound the results. To eliminate the effect of lung function on our data, study participants with and without CFRD were matched based on FEV_1_ % pred. To be considered a match, the difference in FEV_1_ % pred between the paired participants were not allowed to exceed five percentage points. This resulted in one group with CFRD (n=10) and one group without CFRD (n=10) with similar lung function (median FEV_1_ 72% pred (IQR 61–82), *versus* 73% pred (62–86), p=0.75) (supplementary figure S1A). The two groups were also comparable in age (supplementary figure S1B) and *P. aeruginosa* colonisation (70% *versus* 60%, p>0.99). When comparing inflammatory markers in the lung function matched groups, there were no significant differences in sputum levels of NE, inflammatory cytokines or total bacterial load between individuals with and without CFRD, although there was a non-significant trend towards higher IL-1β levels in the CFRD group (median 373 ng·mL^−1^ (IQR 152–1421), *versus* 2080 ng·mL^−1^ (340–6724), p=0.11) (supplementary figure S1C-I).

Taken together, CFRD is associated with increased airway inflammation, but the strong association between impaired glucose control and low lung function makes it difficult to determine whether the increased airway inflammation is caused by CFRD or if it is a marker of low lung function.

### Sputum proteome analyses reveal distinct protein profiles associated with CFRD

**FIGURE 2:**
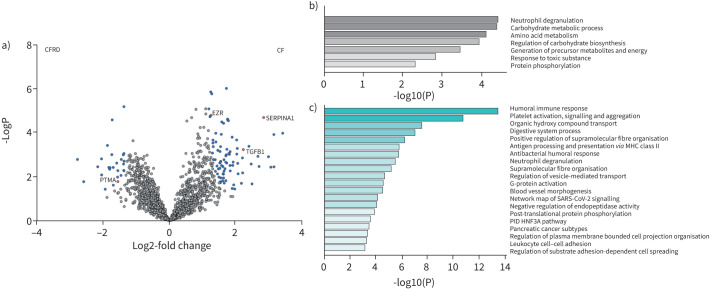
Sputum proteins significantly associated with cystic fibrosis (CF)-related diabetes (CFRD). a) Volcano plot showing proteins significantly associated with CFRD and non-CFRD. Statistical significance was determined using a two-tailed t-test with an FDR of 0.05 to correct for multiple comparisons. Coloured dots (blue and red) represent proteins significantly associated with CFRD or non-CFRD. b–c) Gene enrichment analyses of the 27 proteins significantly associated with CFRD (b) and 83 proteins associated with non-CFRD (c) using Metascape.

To explore patterns of inflammation in relation to CFRD in more detail, the total sputum proteome was analysed using DIA LC-MS/MS. In total, 2313 human proteins identified by at least two peptides were found across all samples (supplementary table S1). Comparing proteomes of pwCF with and without CFRD, 27 proteins were found to be significantly associated with CFRD and 83 were associated with non-CFRD samples (figure 2a and supplementary table S2). Further gene enrichment analyses using Metascape revealed that the most enriched biological processes of the 27 proteins associated with CFRD were neutrophil degranulation and carbohydrate metabolic processes, suggesting a possible connection to inflammation and glucose metabolism (figure 2b). In comparison, the 83 proteins associated with no CFRD were primarily involved in humoral immune responses and platelet activation (figure 2c).

**FIGURE 3:**
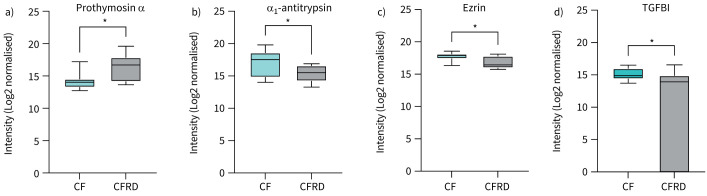
Abundancies of sputum proteins associated with cystic fibrosis (CF)-related diabetes (CFRD) or non-CFRD in patients matched on lung function. Protein abundancies in the sputum proteome from people with CF with and without CFRD in the lung function-matched cohort. The graphs show Log2-normalised intensities in sputum of a) prothymosin α, b) α_1_-antitrypsin, c) ezrin and d) TGFBI. *p<0.05.

We next compared proteomes between pwCF with and without CFRD that were matched on lung function. In a protein enrichment analysis, no proteins were significantly associated with either CFRD or no CFRD (supplementary figure S2). However, when we specifically analysed the abundances of the 27 proteins associated with CFRD in the total cohort, we found that 10 proteins were significantly more abundant in the CFRD group of the lung function-matched cohort (supplementary figure S3). These included prothymosin α (UniProt ID: P06454), which has been implicated to play a role in diabetes and insulin release [25] (figure 3a). Similarly, of the 83 proteins associated with no CFRD in the total cohort, 27 were significantly more abundant in pwCF without CFRD in the lung function-matched group (supplementary figure S4). These included ezrin (P15311) and transforming growth factor-β-induced protein ig-h3 (TGFBI, Q15582) that have been implicated to have a protective role in diabetes [26, 27], as well as α_1_-antitrypsin (P01009) that is important for maintaining the protease/antiprotease balance in the lower airways [28] (figure 3b–d).

## Discussion

In the present investigation, we demonstrate that CFRD is associated with increased airway NE activity, elevated sputum concentrations of IL-8 and IL-1β, and specific protein patterns in the airway proteome. Furthermore, we report a significantly reduced FEV_1_ % pred in pwCF with CFRD. This study is to our knowledge the first to characterise airway inflammation and the total airway proteome in sputum from adult pwCF with and without CFRD.

Consistent with our findings, several previous studies have described a strong association between CFRD and low lung function [6, 29]. Additionally, increased airway neutrophils and IL-8 were found in bronchoalveolar lavage fluid during elevated plasma glucose levels in young children with CF [30]. In contrast to IL-8 and IL-1β, sputum concentrations of IL-6 were surprisingly lower in our CFRD group compared to pwCF without CFRD. The mechanisms underlying these low IL-6 levels are not fully understood, and it has been described as a “mysterious dichotomy” by Fuchs *et al.* [31]. One proposed explanation is the proteolytic degradation of IL-6 by serine proteases [32]. Moreover, both IL-6 and IL-1β are involved in macrophage stimulation, metabolic stress and glucose homeostasis [33]. Crosstalk between these two cytokines may contribute to our finding of reduced IL-6 but highly elevated IL-1β.

A strength of the present study is the comprehensive mapping of the airway proteome in pwCF with and without CFRD. Using proteomic analyses, we identified distinct proteins and biological pathways associated with CFRD. Specifically, four proteins with direct implications in diabetes and lung function were identified among the differentially expressed proteins: prothymosin-α, α_1_-antitrypsin, ezrin and TGFBI. Prothymosin-α, which was associated with CFRD, has been identified as a genetic modifier of CFRD and is also involved in inflammation, oxidative stress, cell proliferation and apoptosis [25]. Increased serum levels of prothymosin-α have been reported in patients with type 2 diabetes [34], and the protein may affect insulin sensitivity by acting as a ligand for Toll-like receptor 4 (TLR4) [35]. In the airways, prothymosin-α contributes to the pathogenesis of pulmonary emphysema [36, 37]. In pwCF without CFRD, two proteins implicated in diabetes, ezrin and TGFBI, were significantly enriched. Ezrin was downregulated in mouse models of diabetes, and low expression was associated with an altered bronchial epithelial barrier *in vitro* [26]. TGFBI was demonstrated to promote pancreatic islet survival and function, and to attenuate type 1 diabetes development *via* immunoregulatory mechanisms in mouse models of diabetes [27, 38]. Moreover, α_1_-antitrypsin was significantly enriched in the sputum proteome of pwCF without CFRD. α_1_-antitrypsin is an antiprotease that protects the lower airways from harmful effects of proteases such as NE, and it is well established that an imbalance between proteases and antiproteases contributes to airway disease [28]. Interestingly, low levels of α_1_-antitrypsin have been associated with both type 1 and type 2 diabetes [39–41]. In the present study, increased NE activity in the CFRD group (figure 1a) in combination with low levels of antiproteases suggest a possible aggravated protease/anti-protease imbalance in CFRD. Taken together, the proteome analysis revealed proteins and cellular pathways specific to CFRD, many of which have a direct or indirect role in diabetes and inflammatory processes. The results may identify new therapeutic targets for CFRD and CF airway disease. For example, α_1_-antitrypsin has been suggested as a target for therapeutic development in CF [42].

Important limitations of our study include the small sample size, the single centre study design and lung function differences between the groups. The CFRD group had a lower FEV_1_ % pred, which reflects the clinical presentation and natural disease progression of CF. Importantly, increased airway inflammation is associated with disease severity in pwCF [43, 44] and could thus confound our results. In particular, NE activity has been demonstrated to correlate with FEV_1_ % pred [24, 45, 46]. Indeed, no significant differences in NE activity or inflammatory cytokines were detected when we matched pwCF with and without CFRD based on lung function. This, however, may be explained by a small sample size and lack of power. Owing to the strong association between CFRD and low lung function, there is also a risk that relevant biological processes are lost when the groups are matched on lung function. Moreover, additional factors could influence airway inflammation, such as concomitant medication including *CFTR* modulators, adherence to treatment and the composition of the total airway microbiome. Despite the low number of patients, we could still demonstrate proteins with significantly different abundances between pwCF with and without CFRD (figure 3 and supplementary figures S3 and S4). However, larger studies are needed to confirm the findings and to adjust for potential confounding factors.

### Conclusion

Our study demonstrates for the first time that CFRD is associated with increased airway inflammation and specific protein expression patterns. Although airway inflammation is tightly interconnected with both CFRD and low lung function, proteome analyses could identify cellular pathways and sputum proteins associated with CFRD. Thus, this study provides novel insights into the association between CFRD and low lung function. Further research in larger patient cohorts is needed to establish the inflammatory pathways in CFRD-associated lung disease and to minimise confounding effects.

## Supplementary material

**Please note:** supplementary material is not edited by the Editorial Office, and is uploaded as it has been supplied by the author. Supplementary figures 00290-2025.SUPPLEMENT Table S1 00290-2025.SUPPLEMENT Table S2 00290-2025.SUPPLEMENT2
