# Supplementary material for: Characterisation of airway inflammation and proteomes associated with cystic fibrosis-related diabetes
Source: ERJ Open Res. 2025 Nov 10;11(6):00290-2025. doi: 10.1183/23120541.00290-2025 (PMC12598600; doi:10.1183/23120541.00290-2025)
Supplement: Supplementary file 1 [file 00290-2025.SUPPLEMENT.pdf]

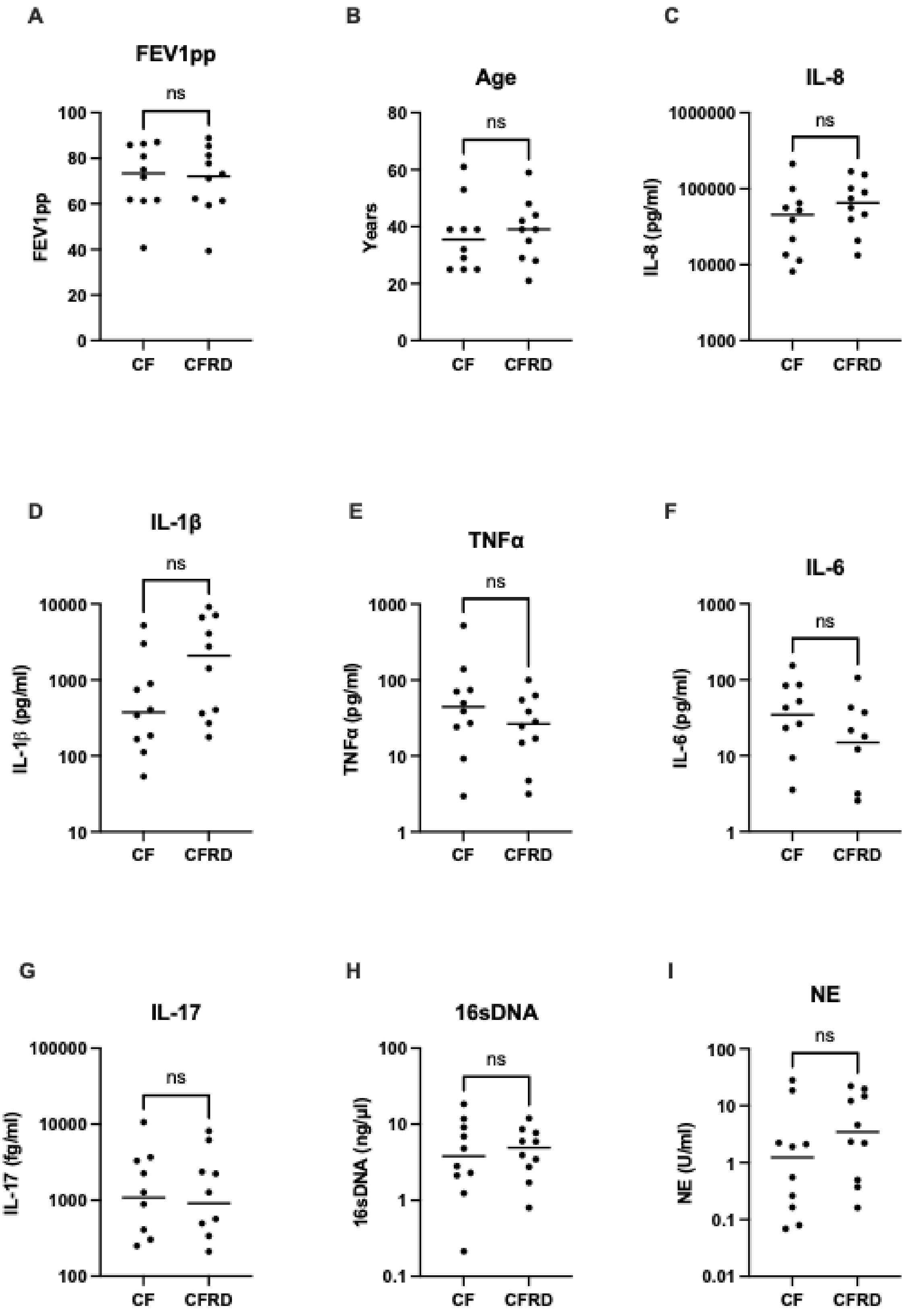

Supplementary Figure 1. Clinical characteristics and expression of inflammatory cytokines in a lung-function matched cohort. Ten pwCF without CFRD were matched with

ten pwCF with CFRD based on FEV1pp (A). The lung-function matched cohort was compared regarding age (B). Sputum levels of IL-8 (C), IL-1β (D), TNFα (E) IL-6 (F), and IL-17 (G) were analysed using Mesoscale U-PLEX assays, bacterial DNA using qPCR of the 16SDNA gene (H) and Neutrophil elastase activity using a chromogenic assay (G). Bars represent median values. ns = not significant.

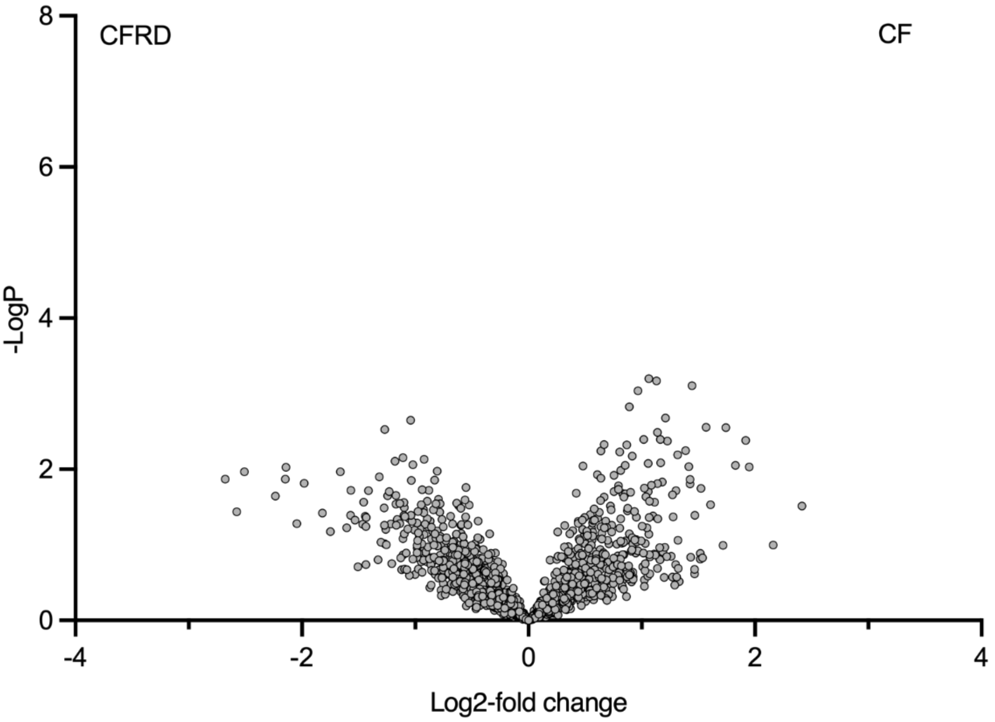

Supplementary Figure 2. Sputum proteins associated with CFRD or non-CFRD in the lung-function matched cohort. Volcano plot showing proteins associated with CFRD and non-CFRD in the lung-function matched cohort. Statistical significance was determined using a both side T-test with an FDR of 0.05 to correct for multiple comparisons. There was no statistically significant association to CFRD or non-CFRD.

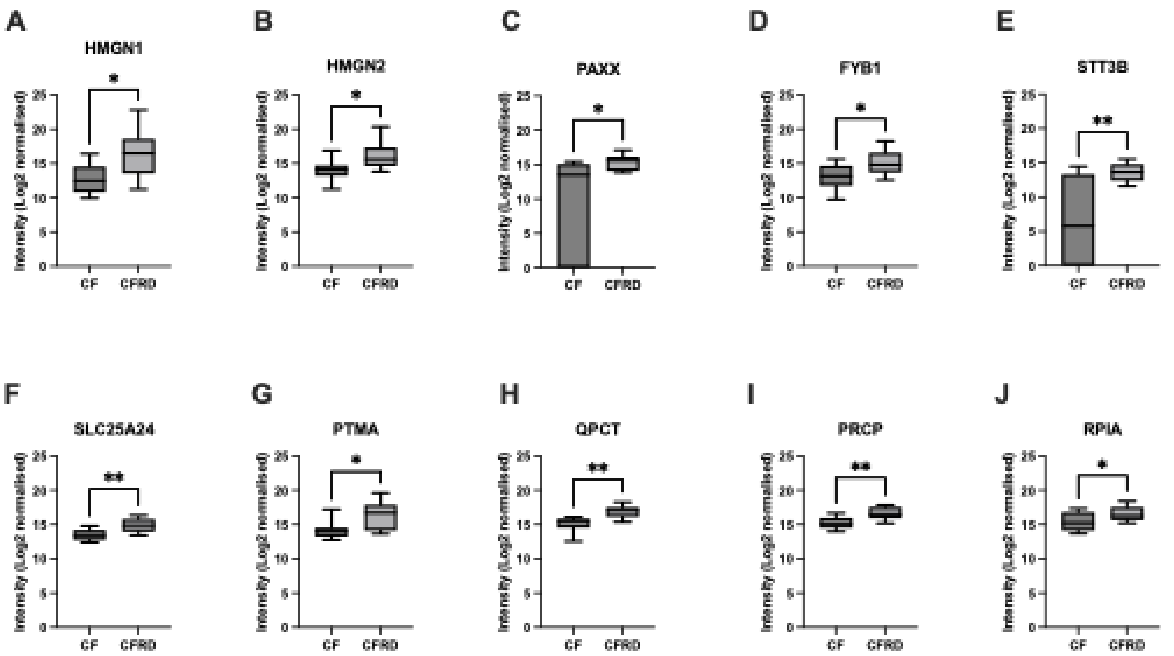

Supplementary Figure 3. Sputum proteins that were significantly more abundant in the CFRD lung-function matched cohort. (A) Non-histone chromosomal protein HMG-14 (HMGN1, UniprotID P05114), (B) Non-histone chromosomal protein HMG-17 (HMGN2, UniprotID P05204), (C) Protein PAXX (PAXX, UniprotID Q9BUH6), (D) FYN-binding protein 1 (FYB1, UniprotID O15117), (E) Dolichyl-diphosphooligosaccharide--protein glycosyltransferase subunit (STT3B, UniprotID Q8TCJ2), (F) Mitochondrial adenyl nucleotide antiporter (SLC25A24, UniprotID Q6NUK1), (G) Prothymosin alpha (PTMA, UniprotID P06454), (H) Glutaminyl-peptide cyclotransferase (QPCT, UniprotID Q16769), (I) Lysosomal Pro-X carboxypeptidase (PRCP, UniprotID P42785), (J) Ribose-5-phosphate isomerase (RPIA, UniprotID P49247). *=p<0.05, **=p<0.01.

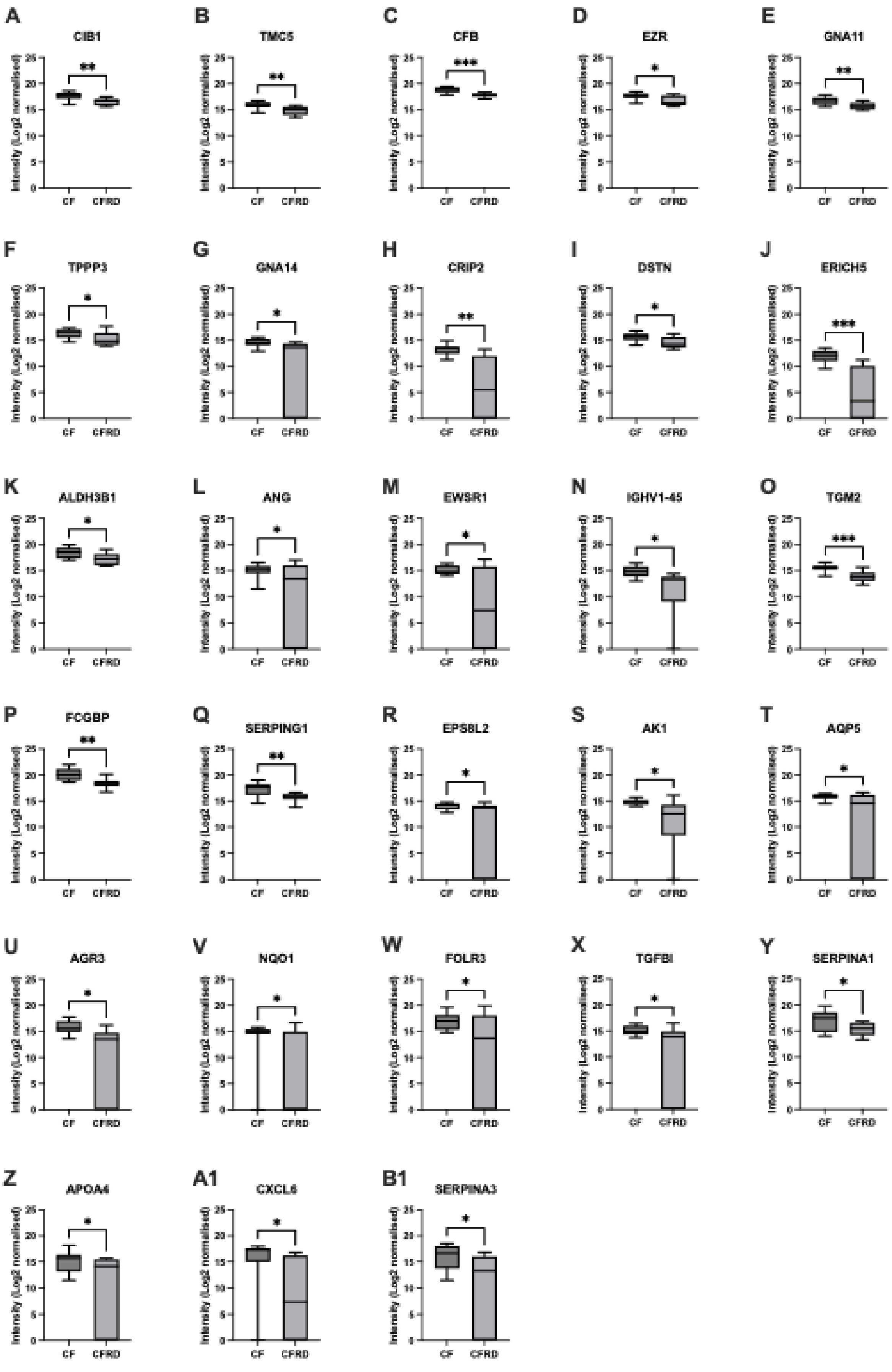
A
CIB1
**
B
TMC5
**
C
CFB
***
D
EZR
*
E
GNA11
**
F
TPPP3
*
G
GNA14
*
H
CRIP2
**
I
DSTN
*
J
ERICH5
***
K
ALDH3B1
*
L
ANG
*
M
EWSR1
*
N
IGHV1-45
*
O
TGM2
***
P
FCGBP
**
Q
SERPING1
**
R
EPS8L2
*
S
AK1
*
T
AQP5
*
U
AGR3
*
V
NQO1
*
W
FOLR3
*
X
TGFBI
*
Y
SERPINA1
*
Z
APOA4
*
A1
CXCL6
*
B1
SERPINA3
*
Intensity (Log2 normalised)
0
5
10
15
20
25
CF
CFRD

Supplementary Figure 4. Sputum proteins that were significantly more abundant in the non-CFRD lung-function matched cohort. (A) Calcium and integrin-binding protein 1 (CIB1, UniprotID Q99828), (B) Brain-specific angiogenesis inhibitor 1-associated protein 2 (TMC5, UniprotID Q6UXY8), (C) Complement factor B (CFB, UniprotID P00751), (D) Ezrin (EZR, UniprotID P15311), (E) Guanine nucleotide-binding protein subunit alpha-11 (GNA11, UniprotID P29992), (F) Tubulin polymerization-promoting protein family member 3 (TPPP3, UniprotID Q9BW30), (G) Guanine nucleotide-binding protein subunit alpha-14 (GNA14, UniprotID O95837), (H) Cysteine-rich protein 2 (CRIP2, UniprotID P52943), (I) Destrin (DSTN, UniprotID P60981), (J) Glutamate-rich protein 5 (ERICH5, UniprotID Q6P6B1), (K) Aldehyde dehydrogenase family 3 member B1 (ALDH3B1, UniprotID P43353), (L) Angiogenin (ANG, UniprotID P03950), (M) RNA-binding protein EWS (EWSR1, UniprotID Q01844), (N) Immunoglobulin heavy variable 1-45 (IGHV1-45, UniprotID A0A0A0MS14), (O) Protein-glutamine gamma-glutamyltransferase 2 (TGM2, UniprotID P21980), (P) IgGFc-binding protein (FCGBP, UniprotID Q9Y6R7), (Q) Plasma protease C1 inhibitor (SERPING1, UniprotID P05155), (R) Epidermal growth factor receptor kinase substrate 8-like protein 2 (EPS8L2, UniprotID Q9H6S3), (S) Adenylate kinase isoenzyme 1 (AK1, UniprotID P00568), (T) Aquaporin-5 (AQP5, UniprotID P55064), (U) Anterior gradient protein 3 (AGR3, UniprotID Q8TD06), (V) NAD(P)H dehydrogenase [quinone] 1 (NQO1, UniprotID P15559), (W) Folate receptor gamma (FOLR3, UniprotID P41439), (X) Transforming growth factor-beta-induced protein ig-h3 (TGFBI, UniprotID Q15582), (Y) Alpha-1-antitrypsin (SERPINA1, UniprotID P01009), (Z) Apolipoprotein A-IV (APOA4, UniprotID P06727), (A1) C-X-C motif chemokine 6 (CXCL6, UniprotID P80162), (B1) Alpha-1-antichymotrypsin (SERPINA3, UniprotID P01011). *=$p<0.05$, **=$p<0.01$.
